# The antioxidant activity, preliminary phytochemical screening of *Zingiber zerumbet* and antimicrobial efficacy against selective endodontic bacteria

**DOI:** 10.1002/fsn3.3462

**Published:** 2023-06-01

**Authors:** Ali A. Assiry, Naveed Ahmed, Abdulmajeed Almuaddi, Ahmed Saif, Mohammed Abdulrahman Alshahrani, Roshan Noor Mohamed, Mohmed Isaqali Karobari

**Affiliations:** ^1^ Preventive Dental Science Department, Faculty of Dentistry Najran University Najran Saudi Arabia; ^2^ Department of Medical Microbiology and Parasitology, School of Medical Sciences University Sains Malaysia Kota Bharu Malaysia; ^3^ Department of Periodontics and Community Dental Sciences, Faculty of Dentistry King Khalid University Abha Saudi Arabia; ^4^ Department of Clinical Laboratory Sciences, Faculty of Applied Medical Sciences Najran University Najran Saudi Arabia; ^5^ Department of Pediatric Dentistry, Faculty of Dentistry Taif University Taif Saudi Arabia; ^6^ Department of Restorative Dentistry & Endodontics, Faculty of Dentistry University of Puthisastra Phnom Penh Cambodia; ^7^ Department of Conservative Dentistry & Endodontics, Saveetha Dental College and Hospitals Saveetha Institute of Medical and Technical Sciences Chennai India

**Keywords:** antibacterial activity, biotechnology, dental, endodontics, ginger extract, MDR pathogens

## Abstract

Antibiotic resistance is rising across the world. For a very long time, bitter ginger (*Zingiber zerumbet*) has been used as one of the most popular herbal remedies to treat a wide range of common diseases. Ginger has been shown to have antioxidant and antibacterial activity. It has various bioactive chemicals that might be utilized as an alternative treatment option for many infectious diseases. The present study aimed to examine the biochemical profile of ginger, antioxidant, and antibacterial activity against selective endodontic microbes. Antioxidant was measured using DPPH and antibacterial activity was performed using disk diffusion tests. *Streptococcus mutants*, *Enterococcus faecalis*, *Staphylococcus* spp., and *Lactobacillus* spp. were tested for antibacterial activity. Before evaluating the dried extracts, all solvents were eliminated using rotary evaporation. The obtained IC50 value revealed that ethanol extract had the greatest antioxidant activity. Concerning each bacterium, the plant extracts demonstrated considerable antibacterial activity (*p* = .001). Ethanol extracts showed the strongest antibacterial activity against the studied microorganisms. This study highlights that the *Zingiber zerumbet* (*Z. zerumbet*) is a strong antibacterial herb against multidrug‐resistant (MDR) gram‐positive bacteria. It may also be employed as a possible natural antioxidant source.

## Graphical abstract

The present study aimed to examine the biochemical profile of ginger, antioxidant, and antibacterial activity against selective endodontic microbes. This study highlights that the *Zingiber zerumbet* (*Z. zerumbet*) is a strong antibacterial herb against multidrug‐resistant (MDR) gram‐positive bacteria. It may also be employed as a possible natural antioxidant source.

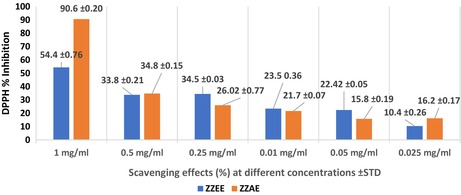

## 1 INTRODUCTION

*Zingiber zerumbet (bitter ginger)* is considered one of the richest sources of secondary metabolites having medicinal potential and is used in Ayurveda, Unani, and Chines medicine (El‐Hack et al., 2020; Mahboubi, 2019). It is used to treat various ailments such as nausea, digestive aid, rheumatism, fever, microbial infection, and bleeding disorder due to a wide variety of volatile oils containing zingiberol, monoterpene, sesquiterpene, and sesquiterpene hydrocarbons (Banerjee et al., 2011). Clinicians and dietitians exceedingly suggest Rhizome as a home remedy against multiple health issues (Pawar et al., 2011; Tariq et al., 2020). Zingberones, shogoals, paradols, and gingerdiols are major phytoconstituents that have been point by point for their antioxidant, anti‐inflammatory, antihyperglycemic, immunomodulatory, anticancer, and cardioprotective properties (Ali et al., 2018; Ramzan et al., 2022).

*Zingiber zerumbet* (L.) Roscoe ex Sm. is the official scientific name for bitter ginger (Prakash et al., 2011). It is a member of the Zingiberaceae family, the biggest family in the Zingiberales order, which has more than 1300 members (Kress et al., 2002). This family member is employed in traditional medicine, agriculture, culinary condiments, and ornamentation, which includes *Zingiber officinale* (ginger; Ahmed et al., 2022), *Curcuma longa* (turmeric; Grzanna et al., 2005), *Zingiber zerumbet* (bitter ginger; Ramzan et al., 2022), and *Elettaria cardamomum* (cardamom; Hemeg et al., 2020). Sesquiterpenoids, flavonoids, aromatic compounds, vanilline, zerumbone, and other polyphenolic compounds are reported in *Zingiber zerumbet* (*Z. zerumbet*; Chang et al., 2012; Koga et al., 2016). All these substances have strong antioxidant activity. Another substance found in bitter ginger called zerumbone is a powerful antioxidant agent and also has anticancer properties (Liu et al., 2017). Several illnesses and other clinical complications, for example arthritis, pain, viral or microbial infections, anti‐aging, gastroenteritis, gastric ulcer, gout, anti‐inflammatory, cancer, diabetes, skin problems, and anti‐allergic have been treated using extracts of bitter ginger (rhizome; Haque & Jantan, 2017).

The antioxidant activity of bitter ginger mainly comes from its phenolic compounds (Nag et al., 2013). Phytochemicals and antioxidants are known to help prevent degenerative diseases caused by oxidative stress by improving the body's antioxidant status. The antioxidant index is key to determining overall health status (Tariq et al., 2020). Having high content of antioxidants in the body boosts immune systems, fights degenerative diseases, and improves overall health. As many pathological conditions such as neurodegenerative diseases, aging, carcinogenesis, and atherogenesis have been linked with the oxidation of the biological components. The rise in free radicals has also been associated with cell degeneration, specifically in the brain. Free radicals accumulate in cells when the natural antioxidant capabilities of cells diminish, or free radicals accumulate in larger amounts. Consuming food having antioxidant activity helps prevent diseases that arise from the accumulation of free radicals or by oxidation of biomolecules (Sam et al., 2019).

*Zingiber zerumbet* commonly known as bitter ginger has many bioactive compounds such as terpenes, carbohydrates, lipids, phenolic compounds, and flavonoids which play an important role as antioxidants and antibacterial agents (Matkowski, 2008). Hence, it would be of huge interest to utilize the potential of bitter ginger for antimicrobial resistance (AMR) and also as an antioxidant. In the present research, the extracts of bitter ginger were tested for the preliminary phytochemical screening, antioxidant, and antimicrobial activity against selective oral microbes (*Enterococcus faecalis*, *Streptococcus mutants*, *Lactobacillus* spp., and *Staphylococcus* spp.).

## 2 MATERIALS AND METHODS

### 2.1 Plant collection and sample preparation

For extraction of antibacterial contents of *Z. zerumbet*, a fresh sample was purchased from a local vegetable market. The ginger rhizomes were properly cleaned with distilled water after sample collection and then dried in the shade. The Department of Pharmacology, Saveetha Institute of Medical and Technical Science, Saveetha University (SU), where a voucher (HA#270917) of plant species was stored for subsequent reference, made the definitive identification of the plant using its vernacular name. The rhizomes of the ginger samples were carefully washed with sterilized distilled water to remove unwanted particles or dust that may lead to contamination of the final product. After that, it was air‐dried at room temperature (25°C). The plant was ground into a fine powder (500 g) after the drying process.

### 2.2 Preparation of organic extract of *Z. zerumbet*

*Zingiber zerumbet* rhizome powder weighing 10 g was mixed thoroughly with 100 mL of distilled water (1:10) in a 250 mL glass container. A water bath was used to boil the mixture for 30 min at 35°C. The extract was boiled and then cooled to room temperature. The mixture or extract is then filtered by filter paper, kept at 5°C for further tests (Anand et al., 2015), and certain filtrates are evaporated to dryness in a vacuum at 40°C using a rotary evaporator machine. The final extract was kept in a regular glass bottle or an airtight container and kept chilled at 4–8°C for further biological activity tests and phytochemical analysis of the crude extract.

### 2.3 Preliminary phytochemical screening

The conventional phytochemical method was used in a preliminary phytochemical examination of the extract to identify the phytoconstituents found in organic extracts of *Z. zerumbet* rhizome, such as phenolics, flavonoids, terpenoids, saponins, and alkaloids (Cyril et al., 2019).

#### 2.3.1 HPLC analysis

High‐performance liquid chromatography (HPLC) was used to analyze the extracts for qualitative phytochemical screening. The following six standards were examined: sinapic acid, myricetin, gallic acid, kaempferol, chlorogenic acid, and caffeic acid. For analysis, the extracts were run through a 0.45 m syringe filter. The Agilent 1260 quaternary pump was installed in the HPLC analyzer (model 1260, USA). Then utilized a DAD detector. A linear gradient with different flow rates was used. The column was kept at a constant temperature of 25°C. At 280 nm, the chromatograms were captured. A software tool called CHEMSTATION was utilized for data analysis (Mradu et al., 2012).

For the mobile phase of the flavonoids, two solvent solutions were used (3% trifluoroacetic acid, and acetonitrile & methanol). This mixture was then isocratically eluted at a flow rate of 1 mL/min at 30°C to perform the chromatographic separation. A 360 nm wavelength was used for detection (Sultana & Anwar, 2008). Phenolic and flavanol identifications were accomplished by comparing their retention times to those of reference standards. The calibration curves of the standards were used to carry out a quantitative determination.

### 2.4 The antibacterial testings of the bacterial strains

By using the Kirby Bauer disk diffusion technique, bacterial strains of antibacterial activity were evaluated (Ramzan et al., 2022). A fresh culture of bacterial isolates was swabbed onto nutrient agar plates using a sterilized cotton swab. It took 2–3 min for the medium's surface to dry. On the inoculated media plates, the antibiotic disks were placed and incubated at 37°C for 18–24 h. After this period, the plates were taken out from the incubator and tested to measure the zone of inhibition (mm) using a measuring ruler (Ahmed et al., 2022).

#### 2.4.1 The collection, inoculation, and reidentification of bacterial strains

To test the antibiotic susceptibility patterns, the *Enterococcus faecalis*, *Streptococcus mutants*, *Lactobacillus* spp., and *Staphylococcus* spp. were collected. The bacterial strain was subcultured on nutrient agar and nutrient broth to produce fresh bacterial growth. The inoculated media plates were then kept at 37°C for a further 24 h. The bacterial colonies were found after the incubation period utilizing a variety of biochemical tests, including the biliary esculin test, catalase, coagulase, and DNA testing (Ahmed et al., 2019; Parveen et al., 2020).

### 2.5 Antioxidant activity of plant extracts

Superoxide dismutase and catalase methods were employed for the determination of antioxidant activity.

#### 2.5.1 Superoxide dismutase activity

To a labeled microtiter plate, the mixture containing 25 μL of 1 M KH_2_PO_4_, 56.5 μL of 10 mM nitro blue tetrazolium (NBT), 5 μL of 5 mM EDTA, 32.5 μL of 100 mM methionine, 10 μL of 2 mM riboflavin, and 121 μL of the plant sample was added in respective wells and kept at room temperature for 10 min. The absorbance was read at 560 nm on an ELISA plate reader (Shamim & Rehman, 2015).

#### 2.5.2 Catalase activity

To a labeled microtiter plate, the mixture containing 12.5 μL of 1 M KH_2_PO_4_, 31.25 μL of 100 mM H_2_O_2,_ and 206.25 μL of the plant sample was added in respective wells and kept at room temperature for 1 min. The absorbance was at 240 nm on an ELISA plate reader (Shamim & Rehman, 2015).

#### 2.5.3 Antioxidant activity

Using the technique developed by Bhakya et al. (2016) free radical scavenging activity was determined with a slight modification. The antioxidant activity of bitter ginger was determined by using 1,1‐Diphenyl‐2 picrylhydrazyl (DPPH; Hara et al., 2018). For this, 0.1 mM DPPH solution was mixed in 82% ethanol. For the stock solution, the extract (100 mg) was mixed with 1 mL of DMSO (10%). Seven different concentrations were prepared from stock solution, that is, 0.0125, 0.025, 0.05, 0.1, and 1.0 mg/mL in 0.5 mM acetic acid buffer. From this mixture, 200 μL was taken and was analyzed for absorbance was noted by using UV double beam spectrophotometer (HALO DB‐20 Dynamica) at a wavelength of 517 nm in triplicates every 20, 40, and 60 min. Standardization was done with ascorbic acid (1–10 μg/mL). A blank test tube was also prepared with the solvent (water) only. Results were reported in terms of IC50 (mg/mL) values. Low IC50 values indicate high antioxidant activity (Rivero‐Cruz et al., 2020). Blank was prepared with no sample. DPPH was employed as the control, while ethanol served as the blank. The percentage inhibition used to represent *Z. zerumbet's* free radical scavenging activity was calculated using the formula below:

%Radical scavenging activity=A0−A1/A0×100

where, A1, the absorbance of the sample; A0, the absorbance of blank.

### 2.6 Statistical analysis

Data were analyzed using Microsoft Excel (Means and SD) and SPSS software. The mean values and SD were used to define the data. T‐test was used to check the significance of using the antibacterial activity of extracts versus antibiotic susceptibility pattern of the tested isolates and DPPH scavenging activity at different concentrations. ANOVA was applied among and between the extracts and antibiotic susceptibility testing for the selected antibiotic. The *p*‐value of <.05 was taken as significant.

## 3 RESULTS

### 3.1 Phytochemical analysis of *Z. zerumbet*

The phytochemical components in an organic extract of *Z. zerumbet* that reduce and cap silver nanoparticles of zerumbet were qualitatively examined. *Z. zerumbet*'s phytochemical evaluation is demonstrated in Table 1; *Z. zerumbet* extracts revealed a high amount of secondary metabolites.

**TABLE 1:** The qualitative phytochemical screening of organic extract of *Z. zerumbet*.

Sr. No	Test	*Zingiber zerumbet*
Test for tannins and phenolic compound		
1	Gelatin	+
2	Ferric Chloride	+
Test for alkaloid		
3	Mayer	−
4	Wagner	−
Test for flavonoids glycoside		
5	Alkaline reagent	+
Test for saponin glycosides		
6	Saponin	−
Test for steroid and triterpenoids		
7	Salkowski	+
8	Libermann Buchard's	+
Test for phytosterols		
9	Phytosterol	−

*Note*: +, Positive; −, Negative.

### 3.2 HPLC analysis of *Z. zerumbet*

The five phenolic and three flavonoid compounds in the *Z*. *zerumbet* extracts were separated in a total run time of 16 and 10 min, respectively, in the following order: gallic acid, chlorogenic acid, caffeic acid, sinapic acid, benzoic acid, myricetin, quercetin, and kaempferol (shown in Table 2).

**TABLE 2:** Phenolic contents in *Zingiber zerumbet*.

Flavonoid & Phenolics	Retention time	Area (%)	Wavelength (nm)	mg/g
Gallic acid	7.117	2.5695	280	0.173
Chlorogenic acid	9.391	1.8186		0.021
Caffeic acid	10.23	1.1224		0.126
Sinapic acid	11.619	7.5296		0.016
Benzoic acid	12.826	0.2307		0.045
Myricetin	2.941	30.1837	360	0.267
Quercetin	3.424	6.3434		0.083
Kaempferol	4.603	0.4445		0.053

### 3.3 Antimicrobial susceptibility test

Table 3 shows the antibacterial potential of plant extracts. The zone of inhibition was higher when the bacterial isolates were tested without ginger extracts. But when the isolates were tested with ginger extracts, the zone of inhibitions was decreased.

**TABLE 3:** Antibacterial activity of *Zingiber zerumbet* extracts.

Sr. No.	Tested strains	Zone of inhibitions (means and ± SD)		
		ZZEE	ZZAE	CIP
1	*Streptococcus mutants*	18.2 ± 0.28	15.5 ± 0.5	25.5 ± 0.5
2	*Enterococcus faecalis*	10.5 ± 0.5	10.5 ± 0.5	29.2 ± 0.28
3	*Staphylococcus* spp.	10.5 ± 0.5	0.0	25.5 ± 0.5
4	*Lactobacillus* spp.	10.5 ± 0.5	10.5 ± 0.5	10.5 ± 0.5

Abbreviations: CIP, Ciprofloxacin; ZZAE, *Zingiber zerumbet* rhizome aqueous extract; ZZEE, *Zingiber zerumbet* ethanol extract.

The results of the antibacterial activities of bitter ginger (*Z*. *zerumbet*) rhizome crude ethanol and aqueous extract of the rhizome are presented in Table 1. The zones of inhibition produced by the crude ethanol extract, and aqueous extract of ginger ranged from 9.17. to 25.5 mm. The highest antibacterial activity (25.5 mm) was noted against *Enterococcus faecalis*.

The antimicrobial activity of extracts was assessed and quantified by the presence or absence of inhibition zone, zone diameter in mm. The antimicrobial activity of ethanol and aqueous extracts of both were effective against gram‐positive bacterial strains except the aqueous extract of *Z*. *zerumbet* against *Staphylococcus* spp. The highest inhibitory zone was observed against *Enterococcus faecalis* (29.2 ± 0.28) followed by *Streptococcus mutans* (25.5 ± 0.5), *Enterococcus faecalis* (25.5 ± 0.5), and *Lactobacillus* spp. (10.5 ± 0.5 ZZEE).

### 3.4 Antioxidant activity medicinal plant extract

#### 3.4.1 Antioxidant activity plant extract by SOD and catalase method

The antioxidant activity of two medicinal plant part extracts was done by two methods. Table 4 indicates the antioxidant activity when treated with two selected medicinal plant extracts at 1 mg/mL concentration. All plant shows good antioxidant activity. The highest antioxidant activity of ZZAE in the catalase method and ZZEE in the SOD method, respectively.

**TABLE 4:** Antioxidant activities plant extracts using a Catalase method and SOD method.

Plant extract	Catalase method	SOD method
ZZEE	0.14 ± 0.003	0.94 ± 0.02
ZZAE	0.49 ± 0.007	0.79 ± 0.01

Abbreviations: ZZAE, *Zingiber zerumbet* rhizome aqueous extract; ZZEE, *Zingiber zerumbet* ethanol extract.

#### 3.4.2 Antioxidant activity medicinal plant extract

**FIGURE 1:**
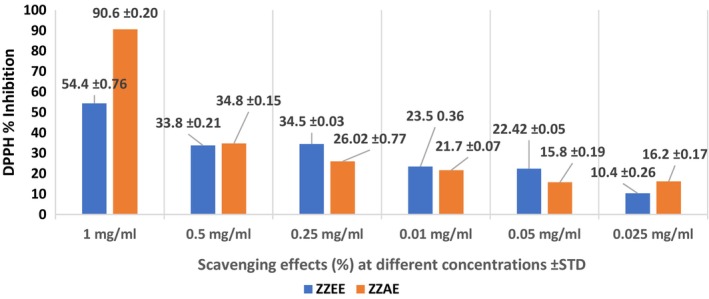
Antioxidant activity plant extracts by DPPH method. ZZAE, *Zingiber zerumbet* rhizome aqueous extract; ZZEE, *Zingiber zerumbet* ethanol extract.

The antioxidant activity of the ginger extract was assessed using the DPPH free radical scavenging test. The above table shows the antioxidant activity of both ginger extracts using DPPH. The positive control was ascorbic acid and the absorbance of all the extracts at different concentrations was compared with it. The highest scavenging activity was present in ZZAE (90.6%) at the highest concentration of 1 mg/mL which is nearest to that of ascorbic acid (95%) at the same concentration. The ZZEE showed less activity (43.9%), respectively, at the same concentration (Figure 1).

## 4 DISCUSSION

The rise in antimicrobial resistance has become a global threat (Rabaan et al., 2022; Zeb et al., 2022). Pathogens of the oral cavity include *Streptococcus mutans*, *Streptococcus* spp., *Staphylococcus* spp., etc. The most common infection of the oral cavity is caused by *S. mutans* which plays a significant role in the formation of dental caries and periodontal disease (Mathai et al., 2017). Although antibiotics like penicillin and vancomycin are found to have excellent anti‐caries effects, their regular use can lead to antibiotic resistance because they alter the oral and intestinal flora. Increasing resistance to common antibiotics has complicated therapy, particularly for multidrug‐resistant organisms (Rabaan et al., 2022). Plants are varied ancient and true natural medicine that is helpful for the treatment of various diseases. According to the World Health Organization (WHO), many people still rely on herbal remedies to treat and maintain their health (Chikezie & Ojiako, 2015). Keeping in mind the scenario, the present research was conducted to evaluate the relative effectiveness of using the extracts of *Z. zerumbet* against MDR pathogens. Many infections have been treated with herbal remedies.

Among the antibacterial effect of these herbal extracts such as bitter ginger, they also have antioxidant effects. So, in the current study, phytochemical analysis of bitter ginger extracts, quantitative, and qualitative analysis of extracts through HPLC, antioxidant activity through DPPH, and antibacterial activity through the disk diffusion method were evaluated. For the quantitative examination of ginger components, gingerols, and shogaols, HPLC is the most trustworthy analytical technique. The HPLC approach is more effective in measuring and identifying phenolic compounds in plants (Ahmed et al., 2022).

Multiple studies have been conducted to identify an efficient alternative strategy for preventing or eliminating E. faecalis from gaining access to the root canal system while the treatment is being administered, in the interim between appointments, or even after the treatment has been finished (Abdollahi‐Mansoorkhani et al., 2022; Islam et al., 2014; O'Hara et al., 1998). Studies have shown that Escherichia coli, Salmonella typhi, and Bacillus subtilis are all susceptible to the antibacterial effects of ginger's active ingredients. Additionally, the ginger ethanolic extract exhibits the largest zone of suppression against S. typhi (Quave wt al., 2008; Sandasi et al., 2010). Antibacterial activity against E. faecalis was highest for 2% CHX, followed by calcium hydroxide and ginger extract in an in vitro study (Kalaiselvam et al., 2019). Based on the findings of a study carried out by Ali et al. (2012) it was determined that an aqueous ginger extract containing 20% can be utilized as a component of endodontic sealer to inhibit the development of bacteria and serve as an effective antibacterial agent.

Ginger is just as effective as ibuprofen in the management of oral diseases and postoperative complications, particularly pain, and it is frequently an adequate replacement for these manufactured agents (Rayati et al., 2017). Ginger was successful in lowering the colony‐forming unit (CFU) of S. mutans to a level that was almost on par with chlorhexidine, the current gold standard for mouthwashes. Ginger‐based rinses were similarly effective at lowering the CFUs/μL of Lactobacillus. In comparison to traditional mouthwash, ginger may be a potential anticarcinogenic and antimicrobial mouthwash with active ingredients that provides a less expensive yet safe caries inhibitory agent (Anushya et al., 2020). The antioxidant properties of ginger and its components have been examined in previous in vivo and in vitro laboratory investigations, which revealed strong antioxidant properties (Ahmed et al., 2022; Ramzan et al., 2022). Moreover, animal tests have shown that ginger extract possesses antioxidant effects. In the current study, the highest free radical scavenging activity (antioxidant activity) was detected for ethanolic extracts. Moreover, the lowest antioxidant activities were detected for the highest concentrations of the extracts. Due to differences in solvent structure and nature, the IC50 values of the tested extracts likely differed. These observations are by a study conducted for extracts of *Z. zerumbet*. According to a study (Khaki & Khaki, 2010), a member of this family significant reduction in the harmful effects of lead acetate exposure on the liver, and oxidative stress was achieved using ginger and its extracts.

The fundamental justification for using bitter ginger was due to its antibacterial characteristics, which implied that ginger itself or its extracts may be used as a cure for diseases with bacteria (Hemeg et al., 2020). Tetracycline, ciprofloxacin, and chloramphenicol were being utilized at the time. The results of this investigation showed that ethanol extracts containing antibiotics were most toxic to *Lactobacillus* species. *n*‐hexane was found to be effective against *E. faecalis* and *S. mutans*. However, chloroform extracts were noted to be effective against *Staphylococcus aureus*. According to a study by Naseer et al (Naseer et al., 2021), bitter ginger's aqueous and ethanolic extracts had similar antibacterial effects on *Staphylococcus aureus* and *Streptococcus pyogenes*.

Dental caries and periodontal infections are the most common dental disorders. Dental caries, which is mostly caused by *S. mutans*, is the most prevalent infectious illness of bacterial origin. The demand for new, innovative antimicrobial medicines or other forms of therapy has been motivated by the growth of MDR bacteria and worries about antibiotic use. The finding of a previous study, showed that ginger was an effective antibacterial agent for gram‐positive bacteria (Sawant et al., 2021). In a previous conducted by Wang et, al ginger essential oils have also been observed to exhibit antibacterial activity against *E. coli* and *S. aureus (*Wang et al., 2020 *)*.

## 5 CONCLUSIONS

According to the present study's findings, *Z. zerumbet* extracts exhibit adequate antibacterial and antioxidant properties for each of their different solvent bases. Drug interaction, toxicity, dosage, and side effects of these extracts along with in vivo interaction and processing (pathophysiological conditions) must be studied as well to better understand their effects. This will make it easier to use these extracts as a treatment or preventive option for many diseases.

## AUTHOR CONTRIBUTIONS

**Ali A. Assiry:** Conceptualization (equal); methodology (equal); visualization (equal); writing – original draft (equal). **Naveed Ahmed:** Formal analysis (equal); methodology (equal); validation (equal); visualization (equal); writing – original draft (equal). **Abdulmajeed Almuaddi:** Validation (equal); visualization (equal); writing – original draft (equal). **Ahmed Saif:** Validation (equal); visualization (equal); writing – original draft (equal). **Mohammed Abdulrahman Alshahrani:** Formal analysis (equal); validation (equal); writing – original draft (equal). **Roshan Noor Mohamed:** Resources (equal); visualization (equal); writing – review and editing (equal). **Mohmed Isaqali Karobari:** Conceptualization (lead); investigation (equal); methodology (lead); project administration (lead); supervision (lead); writing – review and editing (lead).

## FUNDING INFORMATION

This research received no external funding.

## CONFLICT OF INTEREST STATEMENT

The authors declare no conflict of interest.

## INSTITUTIONAL REVIEW BOARD STATEMENT

The study was conducted in accordance with the Declaration of Helsinki and approved by the Institutional Review Board of Saveetha University, Chennai, Tamil Nadu, India, with ethical clearance code: IHEC‐SDC‐FACULTY/21/ENDO/199.

## INFORMED CONSENT STATEMENT

Informed consent was obtained from all subjects involved in the study. Written informed consent has been obtained from the patient(s) to publish this paper.

## Data Availability

The data will be made available on reasonable request to the corresponding author.
